# New Appliances and Things Medical

**Published:** 1905-10-28

**Authors:** 


### NEW APPLIANCES AND THINGS MEDICAL.

[We shall be glad to receive, at our Office, 28 & 29 Southampton Street, Strand, London, W.C., from the manufacturers, specimens of all new preparations and appliances which may be brought out from time to time.]

### CYLLIN PASTILLES.

(Jeyes' Sanitary Compounds Co., Ltd., 64 Cannon Street, London, E.C.)

In a former issue we drew attention to the antiseptic properties of cyllin and noticed the many preparations of this substance. Since that time these preparations have been augmented by a new one—viz. cyllin throat pastilles. The value of throat pastilles in cold, dusty weather is undoubted, and the pastilles before us combine the soothing demulcent action of liquorice and tolu with the antiseptic properties of their active constituent, viz. cyllin. Each pastille contains $\frac{1}{10}$ of a minim of pure cyllin, a quantity adequate to secure a pronounced antiseptic effect and not sufficient to irritate the throat or give a nauseous taste to the pastille. The pastilles are well made, and we have no doubt they will be much appreciated by the public and the profession.

### VALIDOL.

(Zimmer and Co., Frankfurt, Germany.)

We are in receipt of a sample of validol and of some brochures giving the therapeutic properties of this substance. Validol is composed of valerianic acid and menthol. In contrast to menthol this substance is soluble, and is dispensed by the proprietors in small bottles containing 10 grammes by weight. Pharmacologically it combines the properties of valerian and menthol, and is exceedingly useful as a restorative in cases of sudden syncope or collapse, and also in hysteria. The value of valerian in these conditions has been recognised for a very long time, and it is probable that this menthol-valerian compound will have an extensive field of usefulness before it. It does not form a solution in water but can easily be given in water, a warm pleasant sensation in the stomach gradually spreading all over the splanchnic area almost immediately follows its ingestion. The dose is a few drops; it has been found useful in sea-sickness.

